# Post-marketing safety evaluation of mirogabalin using the JADER database

**DOI:** 10.3389/fphar.2026.1833433

**Published:** 2026-06-04

**Authors:** Xinjuan Sun, Liyi Li, Gai Li, Lei Wang, Haitao Zhang, Chenyang Lu, Yu Zhao, Jin’an Chen, Aiping Wang

**Affiliations:** Nanjing University of Chinese Medicine Affiliated Junxie Hospital, Nanjing, China

**Keywords:** diabetic neuropathy, JADER, mirogabalin, neuralgia, pharmacovigilance, postherpetic neuralgia

## Abstract

**Background:**

Mirogabalin, a novel third-generation α2-δ calcium channel ligand, is approved for diabetic peripheral neuropathic pain (DPNP). However, its post-marketing safety profile remains insufficiently characterized, particularly regarding novel signals and comparative safety profiles with established therapies.

**Objectives:**

This study aimed to identify and characterize adverse event (AE) signals associated with mirogabalin, evaluate demographic differences, and compare signal strength with pregabalin and gabapentin.

**Methods:**

We conducted a pharmacovigilance analysis using the Japanese Adverse Drug Event Report (JADER) database (January 2019–June 2025). Disproportionality analysis was performed using the Reporting Odds Ratio (ROR) and Information Component (IC) methods. Subgroup analyses by sex and age, sensitivity analyses restricted to monotherapy reports, and time-to-onset (TTO) analysis using Weibull modeling were conducted.

**Results:**

Among 463 healthcare professional-reported AE reports, 33 positive signals were identified. Beyond known AEs (e.g., dizziness, somnolence), six novel potential signals were detected: pleural effusion (n = 6), contusion (n = 4), toxic encephalopathy (n = 4), Cardiac failure congestive (n = 4), deafness (n = 3), and angioedema (n = 3). Head-to-head comparisons revealed that mirogabalin exhibited significantly higher reporting odds ratios (RORs) than pregabalin for pleural effusion (ROR = 2.39, 95%CI: 1.01–5.65), toxic encephalopathy (ROR = 6.37, 95%CI: 1.99–20.36), contusion (ROR = 3.35, 95%CI: 1.14–9.87), and deafness (ROR = 3.67, 95%CI: 1.04–12.91), and significantly higher RORs than gabapentin for cardiac failure congestive (ROR = 6.05, 95%CI: 1.78–20.54). Subgroup analysis revealed sex- and age-related differences: male patients showed higher reporting proportions of muscle weakness, myoclonus, erythema multiforme, urinary retention, and elevated liver enzymes, whereas females exhibited higher proportions of renal impairment and falls; elderly patients (≥65 years) showed higher reporting frequencies of dizziness and rhabdomyolysis, whereas the 18–65 age group showed higher proportions of hepatic and cardiovascular events. Sensitivity analysis partially supported the stability of some signals, with rhabdomyolysis identified exclusively in monotherapy reports. The median TTO was 6 days (IQR: 2–42), indicating an early-clustering pattern (β = 0.55, 95% CI: 0.50–0.60). Sensitivity analysis after excluding outliers yielded a median TTO of 5 days (IQR: 2–28) with β = 0.72 (95% CI: 0.65–0.80).

**Conclusion:**

This study extends the understanding of mirogabalin’s post-marketing safety profile by identifying six novel signals and revealing distinct safety signal profiles compared with established α2-δ ligands. Demographic-stratified monitoring strategies are warranted to optimize patient safety.

## 1 Introduction

Diabetic peripheral neuropathic pain (DPNP)—characterized by burning, shooting, or tingling sensations—affects a substantial proportion of patients with diabetes, with prevalence increasing alongside disease duration (Tao et al., 2025; Abdissa, 2020; Li et al., 2023). The International Diabetes Federation estimates that 643 million adults worldwide were living with diabetes in 2024, a number projected to reach 853 million by 2050 (Duncan et al., 2025). DPNP imposes significant clinical and economic burden and substantially impairs quality of life.

Calcium channel α2-δ ligands, including gabapentin, pregabalin**,** and other first-line agents, represent the mainstay of pharmacotherapy for DPNP. These agents bind the α2δ subunit of voltage-gated calcium channels, reducing calcium influx and suppressing excitatory neurotransmitter release (glutamate, noradrenaline, substance P) (Wu et al., 2025). Clinical trials consistently demonstrate their efficacy in pain relief and quality-of-life improvement, with somnolence and dizziness reported most frequently (Wiffen et al., 2017; Lesser et al., 2004; Baba et al., 2019).

Mirogabalin, a novel third-generation α2-δ ligand approved in Japan in 2019, has shown promise in neuropathic pain management. However, pivotal trials have primarily characterized short-term safety profiles, and real-world evidence remains scarce—particularly regarding specific patient subgroups and comparative safety signals relative to established therapies (Guo et al., 2024; Xi and Tang, 2025). This study therefore aimed to: (1) identify post-marketing AE signals for mirogabalin using the Reporting Odds Ratio (ROR) and Bayesian Confidence Propagation Neural Network (BCPNN) methods; (2) analyze AE composition across age and sex subgroups; (3) assess signal consistency through sensitivity analyses restricted to monotherapy reports; and (4) characterize time-to-onset distributions using Weibull modeling and compare the signal strength of newly identified events with those of pregabalin and gabapentin to inform safer clinical use.

## 2 Materials and methods

### 2.1 Data source and extraction

All reports for mirogabalin (Japanese generic name: ミロガバリンベシル酸塩; brand name: タリージェ錠/Tarlige®) submitted between January 2019 and June 2025 were extracted from the JADER database, publicly available from the Pharmaceuticals and Medical Devices Agency (PMDA) of Japan (www.pmda.go.jp). The database comprises four tables: Demographic Information (DEMO), Drug Information (DRUG), Adverse Reaction Information (REAC), and Medical History (HIST), which were linked using a unique case identification number.

### 2.2 Data preprocessing and case selection

**FIGURE 1:**
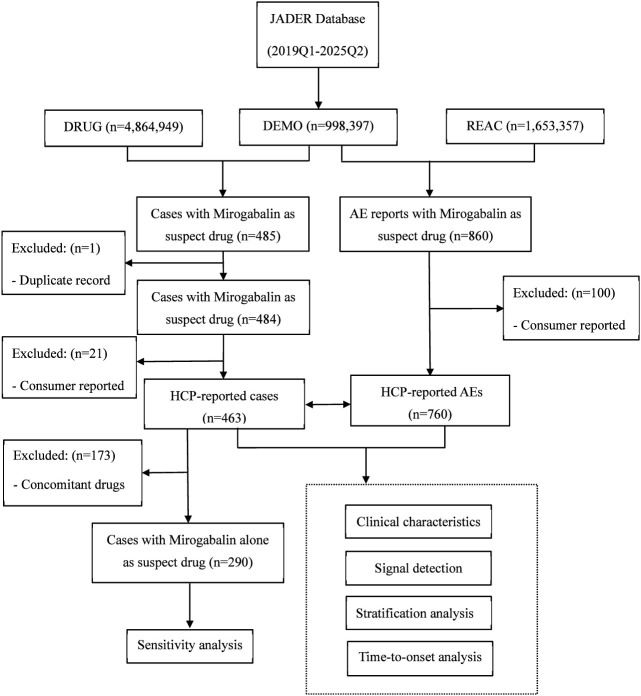
Flowchart of case selection and data processing. Note: DRUG, drug information; DEMO, patient demographic information; REAC, adverse reaction information; AE, Adverse Event; HCP, Healthcare Professional. Deduplication was performed at the case level using the unique patient report number (primaryid). For each case, only the most recent report (maximum ‘number’) was retained. Within each case, the record with the smallest drug sequence number—corresponding to the primary suspected drug (drug_seq = 1)—was selected. Consumer-reported cases and cases with concomitant drugs were excluded to ensure data quality. Sensitivity analysis was performed using cases where mirogabalin was the sole suspected drug.

The JADER database identifies unique cases based on the combination of “patient report number” and “report date.” To avoid duplication, only the latest submitted report for each unique case was retained. In practice, this deduplication process retained the record with the maximum ‘number’ and the primary suspected drug defined by drug_seq = 1. From the DRUG table, only reports where mirogabalin was recorded as the “suspected drug” in the “drug involvement” field were included. Furthermore, to ensure data quality, the analysis was restricted to reports submitted by healthcare professionals (e.g., physicians, pharmacists). AEs were coded to Preferred Terms (PTs) using the Medical Dictionary for Regulatory Activities (MedDRA), version 27.1, and categorized by System Organ Class (SOC). The overall data flow is summarized in Figure 1.

All analyses were stratified by unit of analysis. Case-level analyses summarized demographic characteristics using the number of unique cases as the denominator. AE-report-level analyses calculated outcome proportions using the number of AE reports as the denominator. Drug-event-pair-level analyses were used for disproportionality analysis. PT-level analyses focused on individual preferred terms.

### 2.3 Signal detection

Signals of disproportionate reporting were detected using the Reporting Odds Ratio (ROR) and the Bayesian Confidence Propagation Neural Network (BCPNN). A hierarchical strategy was applied, with thresholds and significance rules summarized in Table 1.

**TABLE 1:** Signal detection thresholds and significance rules.

Analysis level	ROR threshold	BCPNN threshold	Minimum reports	Signal requirement
SOC	Lower 95% CI > 1	IC_025_ > 0	≥1	Either criterion
PT	Lower 95% CI > 1	IC_025_ > 0	≥3	Both criteria required

SOC, system organ class; PT, preferred term; ROR, reporting odds ratio; BCPNN, bayesian confidence propagation neural network; CI, confidence interval; IC_025_, information component with 95% confidence interval lower bound >0. For SOC, level, either ROR (Lower 95% CI > 1) or BCPNN (IC_025_ > 0) is sufficient to signal; for PT, level, both criteria are required. Min Cases: minimum number of reports of the target adverse event with the target drug.

At the SOC level: A signal was considered positive if it met either the ROR criterion or the BCPNN criterion. At the PT level: A signal was confirmed as positive only when it satisfied both the ROR criterion and the BCPNN criterion, with at least three reported cases. The formulas for disproportionality analysis and the definitions of the contingency table are provided in Supplementary Table S1. Novel signals in this study refer to AEs not previously reported in the Japanese PMDA prescribing information for mirogabalin (Tarlige®). The classification of novel signals was based on direct comparison with the events listed in the product label.

It is important to note that signals identified from spontaneous reporting systems indicate a statistical association worthy of further investigation, and do not quantify incidence or absolute risk. Similarly, outcome proportions reported in this study reflect reporting patterns in the pharmacovigilance database and should not be interpreted as drug-attributed incidence or mortality rates. Spontaneous reporting systems are inherently limited by underreporting and missing data.

### 2.4 Subgroup and sensitivity analyses

Subgroup analyses were conducted to explore potential differences in reporting patterns by sex (male, female) and age (18–65 years, ≥65 years). Sensitivity analysis was performed to assess the robustness of the primary signals by restricting the dataset to reports involving mirogabalin monotherapy (i.e., excluding all cases with any concomitant medications). Signal detection was repeated in this subgroup, and findings were compared with the primary analysis.

### 2.5 Novel signal outcomes and comparative analysis

For the identified novel signals, we listed the number of reports and their outcomes. To contextualize the signal strength, we further compared the ROR values between mirogabalin and established α2-δ ligands (pregabalin and gabapentin) for the same AEs.

### 2.6 Time-to-onset (TTO) analysis

Time-to-onset (TTO) was calculated as the interval (in days) between the drug administration date and the AE onset date. Only reports with complete and valid date information were included. Negative intervals (TTO < 0) and implausible intervals (e.g., TTO > 365 days, based on clinical expectation of acute AE occurrence) were excluded. Reports with missing or ambiguous dates (e.g., only year or month provided) were also excluded. For AEs occurring on the day of drug initiation, TTO was recorded as 0. The median and interquartile range (IQR) were calculated based on the original TTO data. A constant offset of 0.5 days was added to all TTO values to accommodate the Weibull distribution’s requirement for positive values (t′ = t + 0.5) (Ando et al., 2019). The Weibull distribution was fitted to the TTO data using maximum likelihood estimation (R package fitdistrplus). The shape parameter (β) and its 95% CI were estimated. β < 1 suggests an early reporting cluster, indicating that AEs are more likely to be reported shortly after drug initiation. This highlights the importance of early monitoring during the initial treatment phase, although it may also reflect reporting biases inherent to spontaneous reporting systems rather than a true decrease in biological risk over time; β ≈ 1 suggests a random failure type (constant hazard), and β > 1 suggests a wear out failure type (hazard increasing over time). Analyses were conducted at both the MedDRA PT and SOC levels. Sensitivity analyses were performed by excluding outliers defined by the interquartile range (IQR) method (Q1 − 1.5 × IQR, Q3 + 1.5 × IQR) to test the robustness of the results.

### 2.7 Statistical analysis

Data management, preprocessing, and disproportionality analysis were performed in R (version 4.3.2; R Foundation for Statistical Computing), primarily through the RStudio integrated development environment (version 2023.12.1+402). Subgroup comparisons of reporting proportions were performed using appropriate statistical tests. Fisher’s exact test was applied to contingency tables containing zero or small expected cell counts, while the chi-square test was applied to tables with adequate cell counts. All statistical tests, including subgroup analyses, were conducted using SPSS (version 27.0; IBM Corp.), with a two-sided P < 0.05 considered statistically significant. Continuous data are presented as median (interquartile range, IQR), and categorical data as counts (percentages).

## 3 Results

### 3.1 Case selection and baseline characteristics

Between January 2019 and June 2025, a total of 485 AE reports with mirogabalin as the suspected drug were identified from the JADER database. After deduplication, 484 unique PS cases remained (1 duplicate record removed). After excluding consumer-reported cases, 463 healthcare professional-reported cases were included in the analysis at the case level. One duplicate record was removed, which had negligible impact on signal detection, outcome proportions, and TTO analysis.

Among the 463 cases, female patients predominated 56.2% (260/463). In terms of clinical indications, neuralgia 17.9% (83/463), diabetic peripheral neuropathy 16.6% (77/463), and postherpetic neuralgia 6.9% (32/463) were the top three indications for mirogabalin use. The patient population was predominantly elderly 66.1% (306/463). Detailed baseline characteristics are presented in Table 2.

**TABLE 2:** Baseline characteristics of cases (n = 463).

Characteristics	Number (n)	Proportion (%)
Gender		
Male	186	40.2
Female	260	56.2
Unknown	17	3.6
Age group		
<18	1	0.2
18–65	137	29.6
≥65	306	66.1
Missing	19	4.1
Indications (top 3)		
Neuropathic pain	83	17.9
Diabetic neuropathy	77	16.6
Post-herpetic neuralgia	32	6.9

Data are presented as n and %. Proportions were calculated based on the total number of unique cases (N = 463).

### 3.2 Adverse event outcomes

At the AE level, a total of 760 HCP-reported AE records were included. Regarding clinical outcomes, recovery was the most common endpoint 39.7%, followed by symptom alleviation 21.8%. Notably, 1.8% of patients (14/760) experienced persistent sequelae, and 11.2% (85/760) remained unrecovered. The overall death proportion was 4.5% (34/760), while the proportion of fatal outcomes among reports with documented outcomes was 5.7% (34/601). Detailed outcomes are presented in Table 3.

**TABLE 3:** Characteristics of adverse event records (n = 760).

Characteristics	Number (n)	Proportion (%)
Outcome of AEs		
Recovered with sequelae	14	1.8
Recovered	302	39.7
Recovering	166	21.8
Death	34	4.5
Not recovered	85	11.2
Unknown	159	20.9

Data are presented as n and %. Proportions were calculated based on the total number of AE, records included in the analysis (N = 760).

### 3.3 Signal detection at the SOC level

AEs associated with mirogabalin were classified into 25 SOCs (Table 4). Ear and labyrinth disorders was the only SOC that met both the ROR and BCPNN criteria. The remaining six SOCs met only the ROR or BCPNN criterion: nervous system disorders, renal and urinary disorders, general disorders and administration site conditions, injuries, poisoning and procedural complications, musculoskeletal and connective tissue disorders, and social circumstances.

**TABLE 4:** Signal strength of mirogabalin-associated adverse events at the system organ class level in the JADER database.

System organ class (SOC)	n	ROR (95% two-sided CI)	IC (IC 025)
Nervous system disorders#	197	3.55 (3.02–4.18)	1.53 (−0.14)
Renal and urinary disorders#	76	2.78 (2.19–3.52)	1.38 (−0.29)
General disorders and administration site conditions#	64	1.35 (1.04–1.74)	0.4 (−1.27)
Injury, poisoning, and procedural complications#	45	2.15 (1.59–2.9)	1.05 (−0.62)
Gastrointestinal disorders	45	0.73 (0.54–0.99)	−0.42 (−2.09)
Skin and subcutaneous tissue disorders	44	0.95 (0.7–1.29)	−0.07 (−1.74)
Investigations	37	0.48 (0.34–0.66)	−0.99 (−2.66)
Respiratory, thoracic, and mediastinal disorders	34	0.58 (0.41–0.82)	−0.73 (−2.4)
Musculoskeletal and connective tissue disorders#	31	1.59 (1.11–2.27)	0.64 (−1.03)
Metabolism and nutrition disorders	30	0.89 (0.62–1.28)	−0.16 (−1.83)
Hepatobiliary disorders	29	0.92 (0.63–1.33)	−0.12 (−1.79)
Cardiac disorders	28	0.9 (0.61–1.31)	−0.15 (−1.82)
Psychiatric disorders	20	1.42 (0.91–2.22)	0.5 (−1.17)
Infections and infestations	18	0.28 (0.17–0.44)	−1.77 (−3.44)
Blood and lymphatic system disorders	15	0.28 (0.17–0.46)	−1.78 (−3.45)
Eye disorders	11	1.02 (0.56–1.85)	0.03 (−1.64)
Ear and labyrinth disorders*	10	7.18 (3.84–13.41)	2.82 (1.15)
Vascular disorders	7	0.35 (0.17–0.73)	−1.5 (−3.17)
Immune system disorders	4	0.16 (0.06–0.43)	−2.6 (−4.27)
Surgical and medical procedures	4	1.62 (0.61–4.34)	0.7 (−0.97)
Endocrine disorders	3	0.21 (0.07–0.64)	−2.25 (−3.92)
Neoplasms, benign, malignant, and unspecified (including cysts and polyps)	3	0.09 (0.03–0.29)	−3.37 (−5.04)
Reproductive system and breast disorders	2	0.72 (0.18–2.89)	−0.47 (−2.14)
Pregnancy, puerperium, and perinatal conditions	1	0.34 (0.05–2.38)	−1.57 (−3.24)
Social circumstances#	1	3.5 (0.49–24.92)	(0.13)

* SOC, level signal confirmed by both ROR, and BCPNN, criteria.

# SOC, level signal confirmed by either ROR, or BCPNN, criterion.

### 3.4 Signal detection at the PT level

According to the MedDRA 27.1 classification system, the 463 AE reports with mirogabalin as the suspected drug mapped to 760 PTs. Among these, 111 PTs met the positive signal criteria based on both the ROR and BCPNN methods. After further excluding 78 signals with fewer than three reported cases of specific AEs, a final set of 33 valid PT positive signals was established. Detailed results are described in Table 5.

**TABLE 5:** Signal strength of mirogabalin-associated adverse events at the preferred term level in the JADER database.

PT	n	ROR (95% two-sided CI)	IC (IC 025)
Dizziness	48	20.6 (15.36–27.63)	4.26 (2.59)
Renal impairment	40	5.33 (3.88–7.33)	2.35 (0.68)
Altered state of consciousness	30	8.36 (5.8–12.05)	3.01 (1.34)
Loss of consciousness	20	6.27 (4.02–9.79)	2.61 (0.94)
Fall	18	8.36 (5.23–13.35)	3.03 (1.36)
Drug-induced liver injury	17	5.71 (3.53–9.25)	2.48 (0.81)
Somnolence	16	15.42 (9.38–25.35)	3.91 (2.24)
Muscular weakness	14	12.76 (7.51–21.68)	3.64 (1.97)
Gait disturbance	8	10.24 (5.09–20.58)	3.34 (1.66)
Oedema peripheral	8	8.84 (4.4–17.77)	3.12 (1.45)
Myoclonus	6	31.23 (13.9–70.17)	4.93 (3.26)
Pleural effusion*	6	3.39 (1.52–7.58)	1.75 (0.08)
Oedema	6	6.7 (3–14.99)	2.73 (1.06)
Dysarthria	5	13.86 (5.73–33.5)	3.77 (2.1)
Hepatic enzyme increased	5	8.25 (3.42–19.91)	3.03 (1.36)
Asthenia	4	4.66 (1.74–12.45)	2.21 (0.54)
Contusion*	4	17.82 (6.64–47.81)	4.14 (2.46)
Amnesia	4	28.42 (10.56–76.46)	4.8 (3.12)
Cardiac failure congestive*	4	3.7 (1.38–9.9)	1.88 (0.21)
Toxic encephalopathy*	4	3.75 (1.4–10.02)	1.9 (0.23)
Fracture	3	3.42 (1.1–10.62)	1.77 (0.1)
Dysstasia	3	12.99 (4.16–40.51)	3.68 (2.01)
Deafness*	3	8.24 (2.64–25.65)	3.03 (1.36)
Tremor	3	3.61 (1.16–11.24)	1.85 (0.18)
Dysphagia	3	4.55 (1.46–14.17)	2.18 (0.51)
Generalised oedema	3	7.58 (2.43–23.61)	2.91 (1.24)
Femoral neck fracture	3	10.25 (3.29–31.94)	3.35 (1.67)
Dyslalia	3	17.46 (5.59–54.54)	4.11 (2.43)
Angioedema*	3	5.93 (1.91–18.46)	2.56 (0.89)
Pain	3	3.35 (1.08–10.41)	1.74 (0.07)
Constipation	3	5.02 (1.61–15.64)	2.32 (0.65)
Withdrawal syndrome	3	8.26 (2.65–25.73)	3.04 (1.36)
Gait inability	3	24.51 (7.83–76.7)	4.59 (2.91)

* Novel signals not documented in the product label; Other PT-level signals are listed in the mirogabalin product label.

Beyond the commonly reported AEs in the product label (e.g., dizziness, somnolence, peripheral edema), six novel PT signals were identified: pleural effusion, contusion, Cardiac failure congestive, toxic encephalopathy, deafness, and angioedema.

### 3.5 Subgroup analysis

Subgroup analysis of AE reporting patterns across gender revealed that male patients exhibited a higher reporting proportion of muscle weakness, myoclonus, elevated liver enzymes, erythema multiforme, and urinary retention. In contrast, female patients demonstrated a higher reporting proportion of renal impairment and fall. Age-stratified analysis showed that elderly participants had a higher reporting proportion of dizziness and rhabdomyolysis, whereas the 18–65-year age group presented a higher reporting proportion of drug-induced liver injury, Cardiac failure congestive, amnesia, erythema multiforme, pruritus, constipation, and hypoaesthesia. See Table 6 for detailed findings.

**TABLE 6:** Stratified analysis of mirogabalin-associated adverse events by subgroup.

PT	Sex			Age		
	Male (n = 186)	Female (n = 260)	p	18–65Y (n = 137)	≥65Y (n = 306)	p
Dizziness	15	31	0.186	8	37	***0.044***
Renal impairment	0	27	***<0.001***	10	29	0.455
Altered state of consciousness	14	16	0.568	6	23	0.217
Fall	3	15	***0.028***	3	15	0.181
Somnolence	3	12	0.083	6	10	0.761#
Loss of consciousness	10	10	0.441	6	12	0.821
Drug-induced liver injury	5	11	0.388	11	0	***<0.001*** #
Muscular weakness	10	4	***0.022***	0	11	0.055#
Gait disturbance	4	4	0.724*	0	8	0.128#
Oedema peripheral	4	4	0.724*	0	6	0.184*
Myoclonus	5	0	***0.012*** *	0	6	0.184*
Pleural effusion	0	4	0.144*	NA	NA	-
Contusion	0	4	0.144*	0	3	0.556*
Dysarthria	0	3	0.269*	0	5	0.330*
Oedema	3	3	0.697*	0	4	0.316*
Hepatic enzymes increased	4	0	***0.03*** *	4	0	***0.009*** *
Deafness	NA	NA	-	0	3	0.556*
Amnesia	0	4	0.144*	4	0	***0.009*** *
Cardiac failure congestive	6	3	0.233#	4	0	***0.009*** *
Toxic encephalopathy	0	3	0.269*	0	4	0.316*
Dysstasia	0	3	0.269*	0	3	0.556*
Tremor	NA	NA	-	0	3	0.556*
Dysphagia	NA	NA	-	0	3	0.556*
Generalised oedema	NA	NA	-	0	3	0.556*
Femoral neck fracture	NA	NA	-	0	3	0.556*
Angioedema	3	0	0.072*	0	3	0.556*
Erythema multiforme	4	0	***0.03*** *	3	0	***0.029*** *
Rhabdomyolysis	6	6	0.555	0	12	***0.042*** #
Asthenia	NA	NA	-	0	4	0.316*
Urinary retention	4	0	***0.03*** *	NA	NA	-
Pruritus	NA	NA	-	3	0	***0.029*** *
Constipation	NA	NA	-	3	0	***0.029*** *
Hypoaesthesia	NA	NA	-	3	0	***0.029*** *
Oxygen saturation decreased	NA	NA	-	0	3	0.556*

* Fisher’s exact test.

# Continuity correction test; others, Chi-square test. Subgroup totals may be less than 463 due to missing data (sex unknown: n = 17; age unknown: n = 19).

Bold values indicate statistical significance (p < 0.05).

### 3.6 Sensitivity analysis

After excluding reports involving concomitant medications, 290 reports of mirogabalin monotherapy were retained, documenting a total of 434 AEs with mirogabalin as the sole suspected drug. The analysis revealed that common AEs associated with monotherapy included dizziness, renal impairment, altered consciousness, falls, muscle weakness, gait disturbance, peripheral edema, and drug-induced liver injury. Notably, among the six signals detected in the concomitant medication group, three (pleural effusion, heart failure, and angioedema) persisted in the monotherapy group. Crucially, a unique signal for rhabdomyolysis was identified exclusively in the monotherapy cohort and was not observed among the 33 signals in the concomitant medication group. Detailed findings are presented in Supplementary Table S2.

### 3.7 Comparative signal strength of novel signals

Through pharmacovigilance analysis, six potential novel signals were identified for mirogabalin. Outcomes for these six signals were primarily recovery or improvement, with the exception of pleural effusion, which was associated with two fatal cases (Table 7). To contextualize the signal strength, we performed head-to-head comparisons of these novel signals against established α2-δ ligands. As shown in Table 8, direct comparisons revealed distinct signal strength profiles for mirogabalin relative to pregabalin and gabapentin. Compared with pregabalin, mirogabalin demonstrated significantly stronger signals for pleural effusion (ROR = 2.39, 95% CI: 1.01–5.65), toxic encephalopathy (ROR = 6.37, 95% CI: 1.99–20.36), contusion (ROR = 3.35, 95% CI: 1.14–9.87), and deafness (ROR = 3.67, 95% CI: 1.04–12.91), with 95% CIs excluding 1. Conversely, no significant differences were observed for rhabdomyolysis, Cardiac failure congestive, or angioedema. Compared with gabapentin, a significantly stronger signal was identified only for Cardiac failure congestive (ROR = 6.05, 95% CI: 1.78–20.54).

**TABLE 7:** Clinical course and outcomes of potential novel adverse events associated with mirogabalin.

PT	Cases	Outcomes
Pleural effusion	6	Death (2), recovery (2), improvement (1), unrecovered (1)
Contusion	4	Recovery (2), missing (2)
Cardiac failure congestive	4	Recovery (1), unrecovered (1), missing (2)
Toxic encephalopathy	4	Recovery (1), improvement (2), unrecovered (1)
Deafness	3	Recovery (1), improvement (1), missing (1)
Angioedema	3	Recovery (3)

**TABLE 8:** Head-to-head comparison of reporting odds ratios for adverse events between mirogabalin and pregabalin/gabapentin.

PT	Mirogabalin vs. pregabalin	Mirogabalin vs. gabapentin
	ROR (95%CI)	ROR (95%CI)
Rhabdomyolysis	0.91 (0.51–1.63)	1.02 (0.45–2.33)
Cardiac failure congestive	1.19 (0.74–1.90)	6.05 (1.78–20.54)#
Pleural effusion	2.39 (1.01–5.65)*	5.65 (0.68–47.05)
Angioedema	1.44 (0.44–4.72)	-
Toxic encephalopathy	6.37 (1.99–20.36)*	3.76 (0.42–33.69)
Contusion	3.35 (1.14–9.87)*	-
Deafness	3.67 (1.04–12.91)*	-

* Compared to pregabalin, mirogabalin demonstrated a significantly stronger signal of association with the adverse events of pleural effusion, toxic encephalopathy, contusion, and deafness.

# Compared to gabapentin, mirogabalin exhibited a significantly stronger signal only for cardiac failure, with the remaining adverse events showing either no significant signal or insufficient data for comparison.

### 3.8 Time to onset analysis

Of the 463 AE reports, 228 (49.2%) contained complete and valid date information for both drug initiation and AE onset, and were therefore included in the TTO analysis. The median TTO was 6 days (interquartile range, 2–42 days). Weibull distribution analysis yielded a shape parameter β of 0.55 (95% CI: 0.50–0.60), indicating an early reporting pattern. Sensitivity analyses excluding outliers confirmed the robustness of these results: TTO median of 5 days (IQR, 2–28 days) and β of 0.72 (95% CI: 0.65–0.80). Detailed findings are presented in Table 9. This early reporting pattern was consistently observed across major SOCs, including nervous system disorders, renal and urinary disorders, and general disorders and administration site conditions. In contrast, musculoskeletal and connective tissue disorders exhibited a random failure pattern (β ≈ 1). Detailed SOC-level findings are shown in Supplementary Table S3.

**TABLE 9:** Time to onset and weibull distribution analysis of mirogabalin-associated adverse events.

Drug	Time to onset (days)		Weibull distribution		Type
	n	Median (IQR)	Scale parameter:α (95% CI)	Shape parameter:β (95% CI)	
Mirogabalin	228	6 (2–42)	22.69 (16.96–28.41)	0.55 (0.50–0.60)	Early failure
	212	5 (2–28)	14.76 (11.84–17.67)	0.72 (0.65–0.80)*	Early failure

* Based on the results calculated using a constant offset of 0.5 days.

## 4 Discussion

This pharmacovigilance study analyzed healthcare professional-reported AE reports for mirogabalin from the JADER database. Using dual-method disproportionality analysis, 33 positive safety signals were identified, including six novel potential AEs not previously documented in the product label. Furthermore, sensitivity analyses on monotherapy reports partially supported the stability of some signals, and head-to-head comparisons with pregabalin and gabapentin revealed distinct signal profiles for several serious AEs. Subgroup analyses explored sex- and age-related differences in reporting patterns, revealing notable variations between demographic groups. Notably, elderly female patients constituted the highest proportion of reports, and time-to-onset analysis indicated a distinct early clustering of AEs following treatment initiation. Together, these findings extend the current understanding of mirogabalin’s post-marketing safety profile.

At the SOC level, six safety signals were identified, with nervous system disorders emerging as the most prominent category. Dizziness and somnolence—the most frequently reported AEs in this category—are well-documented in the product label and are consistent with findings from pivotal clinical trials and real-world studies of mirogabalin (Kato et al., 2021; Ushida et al., 2023). The underlying mechanism involves the modulation of voltage-gated calcium channels in the central nervous system, which reduces excitatory neurotransmitter release but may simultaneously cause CNS depression. Notably, our analysis also identified toxic encephalopathy—an unlabeled event within this category. Published evidence suggests that high-dose administration of gabapentin or pregabalin may induce toxic encephalopathy, thereby establishing a plausible class effect that extends to mirogabalin (Sechi et al., 2004; Beauvais et al., 2015; Lee, 2012). Given that mirogabalin exhibits higher binding affinity for the α2δ subunit compared with pregabalin, clinicians should remain vigilant for this rare but serious neurological event, particularly at higher doses or in patients with compromised renal function.

Within the renal and urinary disorders SOC, renal impairment was the sole identified signal—consistent with mirogabalin’s primary route of renal excretion. Similar signals have been documented for pregabalin and gabapentin, supporting a potential class effect (Torregrosa-de Juan et al., 2012; Grunze et al., 1998; Takahashi et al., 2017). These observations underscore the necessity of assessing renal function before and during mirogabalin treatment, particularly in elderly patients who frequently exhibit age-related declines in renal clearance.

Regarding fluid-related events, pleural effusion is not a labeled adverse reaction for mirogabalin, pregabalin, or gabapentin; however, these gabapentinoids share a class effect of fluid retention (e.g., peripheral edema, weight gain), and sporadic case reports have described pleural effusion in association with pregabalin. In our analysis, the significantly stronger signal for mirogabalin versus pregabalin suggests that mirogabalin may carry a distinct pleural effusion signal profile. This is biologically plausible, given that α2-δ ligands may cause vasodilation and fluid redistribution—with additional contributions from sodium retention and renal effects (Largeau et al., 2022). Similarly, angioedema is a labeled adverse reaction for pregabalin and gabapentin (Ortega-Camarero et al., 2012; Chong and Hsu, 2021). In our head-to-head comparison, mirogabalin showed no significant difference from pregabalin, aligning with the known class effect of α2-δ ligands on angioedema reporting. Given the potentially serious consequences—such as laryngeal edema—clinicians should remain vigilant for allergic symptoms in patients receiving mirogabalin.

Cardiac failure congestive represents a newly identified AE not documented in the prescribing information for mirogabalin. Mechanistically, mirogabalin shares a high binding affinity for the α2δ subunits with pregabalin, which has been linked to an increased reporting of cardiovascular AEs. Consistent with this, recent large-scale cohort studies have indicated that gabapentinoid use is associated with increased reporting of major adverse cardiovascular events, including Cardiac failure congestive (Pan et al., 2022; Park et al., 2025). Given this established class effect, the signal detection in our study revealed a significantly stronger association between mirogabalin and “Cardiac failure congestive” compared with gabapentin, suggesting that mirogabalin might pose a distinct signal profile within this drug class. Clinically, enhanced monitoring for signs of fluid retention and cardiac function is advisable, particularly in patients with pre-existing cardiovascular conditions.

Beyond systemic effects, hearing loss is one of the most prevalent diseases worldwide (GBD 2019 Hearing Loss Collaborators, 2021). This study identified deafness as an AE associated with ear and labyrinth disorders; however, no deafness-related AEs have been reported for mirogabalin to date. Literature has documented hearing deterioration in patients receiving pregabalin or gabapentin (Pierce et al., 2008; Yılmaz et al., 2020). Given the increasing clinical utilization of mirogabalin, implementing risk mitigation strategies such as serial audiological assessments is of significant importance in high-risk patients.

Further stratifying the population, this study revealed significant gender- and age-related differences in reporting patterns. Previous studies have reported that pregabalin has higher plasma concentrations in female patients compared with male patients; similarly, female patients receiving gabapentin treatment have a higher likelihood of experiencing AEs (Salis et al., 2024). While these observed gender differences may partly reflect known gender-based reporting tendencies in spontaneous databases, pharmacological factors—such as the potential modulation of neurotransmitter systems by sex hormones—cannot be entirely excluded.

In the age-related reporting pattern, the increased fall events in elderly patients are consistent with the general characteristics of central nervous system drugs. This observation aligns with randomized trial subgroup analyses showing higher dizziness incidence in patients aged ≥65 years receiving mirogabalin, providing a mechanistic basis for the age disparity observed in our real-world data (Tatsumi et al., 2021). Given the exploratory nature of these subgroup findings and the inherent limitations of spontaneous reporting systems, these results should be interpreted as hypothesis-generating rather than definitive clinical guidance. Future targeted safety monitoring might investigate these patterns; for example, by assessing renal function in female patients or focusing on neuromuscular symptoms in male patients. For elderly patients (≥65 years), particular attention to dizziness and musculoskeletal symptoms may inform fall prevention strategies.

Methodologically, sensitivity analysis on monotherapy reports refined the safety signal profile of mirogabalin. The co-occurrence of signals for pleural effusion, cardiac failure congestive, and angioedema in both the monotherapy and concomitant therapy groups suggests potential stability of these signals. Additionally, rhabdomyolysis was detected as a monotherapy-specific signal in the sensitivity analysis—which has been documented as a rare but serious AE of other gabapentinoids (Liu et al., 2025; Zhai et al., 2024)—and was not among the 33 primary signals. This exploratory finding—notably, only three of the six novel signals persisted in the monotherapy analysis—should be interpreted cautiously and warrants further investigation with appropriate clinical validation. This highlights the value of stratified analysis in identifying medication-specific signal patterns.

Despite these strengths, this study has several limitations. The JADER database is a spontaneous reporting system, which entails potential underreporting and reporting biases. The absence of a defined exposed population precludes incidence rate estimation, and limited clinical detail restricts causal inference. Therefore, the identified safety signals are exploratory and hypothesis-generating, requiring validation in well-designed pharmacoepidemiologic studies. Additionally, several newly identified signals were supported by limited case numbers (e.g., n = 3–6 for pleural effusion, cardiac failure congestive, or angioedema). Given the low frequency, these signals may have limited robustness and could reflect spurious associations due to reporting variability inherent to spontaneous reports. Accordingly, such findings should be interpreted cautiously and regarded as hypothesis-generating observations warranting further confirmation.

Furthermore, indication bias represents an important consideration in interpreting our findings. The primary indications for mirogabalin—including neuropathic pain, diabetic peripheral neuropathy, and post-herpetic neuralgia—are inherently associated with older age, falls, renal dysfunction, neurological symptoms, and multiple comorbidities. Consequently, signals such as dizziness, falls, renal impairment, and cardiac failure congestive may partly reflect the underlying disease burden rather than drug-specific effects. While our monotherapy sensitivity analysis partially mitigates this concern by excluding concomitant medications, and our head-to-head comparisons with pregabalin and gabapentin provide contextual evidence, residual indication bias cannot be fully excluded. Future studies with appropriate comparator groups—such as patients with similar indications not receiving gabapentinoids—are warranted to disentangle drug effects from underlying disease effects.

## 5 Conclusion

This large-scale pharmacovigilance study delineates the post-marketing safety profile of mirogabalin, extending beyond pre-approval clinical trials. While validating known AEs (e.g., dizziness, somnolence), we identified six novel potential signals—including pleural effusion, Cardiac failure congestive, and deafness—with distinct signal strengths compared to established α2-δ ligands. Notably, sensitivity analysis revealed rhabdomyolysis as a monotherapy-specific signal, reinforcing the value of stratified analysis. The observed early clustering of AEs, coupled with sex- and age-related reporting disparities, underscores the necessity for demographic-tailored monitoring during the initial treatment phase. These findings provide critical insights for optimizing the safe use of mirogabalin in clinical practice.

## Data Availability

The datasets presented in this study can be found in online repositories. The names of the repository/repositories and accession number(s) can be found in the article/Supplementary Material.
