# Supplementary material for: Post-marketing safety evaluation of mirogabalin using the JADER database
Source: Front Pharmacol. 2026 Jun 4;17:1833433. doi: 10.3389/fphar.2026.1833433 (PMC13275718; doi:10.3389/fphar.2026.1833433)
Supplement: Supplementary file 1 [file Table1.docx]

**Table S1. Computational Framework and Formula Definitions for Disproportionality Analysis**

**1. Two-by-Two Contingency Table**

|  | **Target AEs** | **Others AEs** | **Total** |
| --- | --- | --- | --- |
| mirogabalin | a | b | a+b |
| Non-mirogabalin | c | d | c+d |
| Total | a+c | b+d | N=a+b+c+d |

AEs, adverse events; a, number of cases with target adverse events in the target drug group; b, number of cases with other adverse events in the target drug group; c, number of cases with target adverse events in other drug groups; d, number of cases with other adverse events in other drug groups.

**2. Disproportionality Analysis Formulas**

| **Algorithm** | **Equation** |
| --- | --- |
| ROR | ROR=ad/bc |
|  | 95% CI=e^ln(ROR)±1.96(1/a+1/b+1/c+1/d)^0.5^ |
| BCPNN | IC=log_2_[a(a+b+c+d)/(a+c)(a+b)] |
|  | 95% CI= E(IC) ± 2V(IC)^0.5 |

ROR, Reporting Odds Ratio; BCPNN, Bayesian Confidence Propagation Neural Network; CI, confidence interval; IC, information component; E(IC), the IC expectations; V(IC), the variance of IC.
