# Supplementary material for: Post-marketing safety evaluation of mirogabalin using the JADER database
Source: Front Pharmacol. 2026 Jun 4;17:1833433. doi: 10.3389/fphar.2026.1833433 (PMC13275718; doi:10.3389/fphar.2026.1833433)
Supplement: Supplementary file 2 [file Table2.docx]

**Table S2. Signal Strength of Mirogabalin Monotherapy Reports at the Preferred Term Level in the JADER Database**

| **PT** | **n** | **ROR (95% two-sided CI)** | **IC (IC 025)** |
| --- | --- | --- | --- |
| Dizziness | 34 | 23.68 ( 16.67 - 33.64 ) | 4.45 ( 2.77 ) |
| Renal impairment | 28 | 6.37 ( 4.34 - 9.34 ) | 2.59 ( 0.92 ) |
| Altered state of consciousness | 20 | 9.51 ( 6.07 - 14.9 ) | 3.18 ( 1.51 ) |
| Loss of consciousness | 17 | 8.62 ( 5.3 - 14.01 ) | 3.05 ( 1.38 ) |
| Fall | 13 | 9.97 ( 5.73 - 17.32 ) | 3.27 ( 1.6 ) |
| Muscular weakness | 11 | 16.74 ( 9.19 - 30.49 ) | 4.02 ( 2.35 ) |
| Somnolence | 10 | 16.21 ( 8.65 - 30.38 ) | 3.98 ( 2.31 ) |
| Cardiac failure* | 10 | 3.86 ( 2.06 - 7.24 ) | 1.92 ( 0.25 ) |
| Drug-induced liver injury | 9 | 5.27 ( 2.72 - 10.2 ) | 2.37 ( 0.7 ) |
| Rhabdomyolysis# | 7 | 3.27 ( 1.55 - 6.9 ) | 1.69 ( 0.02 ) |
| Gait disturbance | 6 | 11.72 ( 5.23 - 26.26 ) | 3.53 ( 1.86 ) |
| Oedema peripheral | 6 | 11.46 ( 5.11 - 25.68 ) | 3.5 ( 1.82 ) |
| Myoclonus | 5 | 46.86 ( 19.3 - 113.76 ) | 5.52 ( 3.84 ) |
| Pleural effusion* | 4 | 4.17 ( 1.56 - 11.18 ) | 2.05 ( 0.38 ) |
| Renal failure | 4 | 4.62 ( 1.72 - 12.37 ) | 2.2 ( 0.52 ) |
| Oedema | 4 | 7.88 ( 2.94 - 21.12 ) | 2.96 ( 1.29 ) |
| Fracture | 3 | 5.39 ( 1.73 - 16.79 ) | 2.42 ( 0.75 ) |
| Tremor | 3 | 6 ( 1.93 - 18.7 ) | 2.57 ( 0.9 ) |
| Hepatic enzyme increased | 3 | 9.12 ( 2.92 - 28.42 ) | 3.18 ( 1.5 ) |
| Dyslalia | 3 | 29.72 ( 9.51 - 92.93 ) | 4.87 ( 3.2 ) |
| Angioedema* | 3 | 9.93 ( 3.18 - 30.95 ) | 3.3 ( 1.63 ) |
| Dysarthria | 3 | 13.31 ( 4.27 - 41.51 ) | 3.72 ( 2.05 ) |

* Denotes newly identified adverse event signals. # indicates novel adverse event signals identified in mirogabalin monotherapy.
