# Supplementary material for: Post-marketing safety evaluation of mirogabalin using the JADER database
Source: Front Pharmacol. 2026 Jun 4;17:1833433. doi: 10.3389/fphar.2026.1833433 (PMC13275718; doi:10.3389/fphar.2026.1833433)
Supplement: Supplementary file 3 [file Table3.docx]

**Table S3. Time to Onset, Descriptive Statistics, and Weibull Distribution Parameters of Mirogabalin-Associated Adverse Events by System Organ Class**

| **SOC** | **Time to onset(days)** | | | **Weibull Distribution** | | **Type** |
| --- | --- | --- | --- | --- | --- | --- |
|  | **n** | **Median (IQR)** | **Min-Max** | **Scale Parameter**  **α(95%CI)** | **Shape Parameter**  **β(95%CI)** |  |
| Nervous system disorders | 65 | 2(1-9) | 0-826 | 11.64(5.23-18.05) | 0.47(0.39-0.55) * | Early Failure |
| Renal and urinary disorders | 27 | 8(3-35.5) | 0-465 | 25.58(7.45-43.71) | 0.57(0.41-0.72) * | Early Failure |
| General disorders and administration site conditions | 16 | 10(4.75-60.75) | 0-321 | 33.19(7.89-58.49) | 0.68(0.43-0.93) * | Early Failure |
| Musculoskeletal and connective tissue disorders | 16 | 4.5(2.75-10) | 1-85 | 8.89(3.12-14.66) | 0.80(0.54-1.07) | Random Failure |

* Analysis based on a 0.5-day offset per data point to correct for onset times of zero.
